# Supplementary material for: General practitioners and sales representatives: Why are we so ambivalent?
Source: PLoS One. 2022 Jan 24;17(1):e0261661. doi: 10.1371/journal.pone.0261661 (PMC8786166; doi:10.1371/journal.pone.0261661)
Supplement: S1 Checklist — (PDF) [file pone.0261661.s001.pdf]

No	Item	Guide questions/description	Paragraph	Commentary
		Table I. Consolidated criteria for reporting qualitative studies (COREQ): 32-item checklist		
Domain 1: Research team and reflexivity				
Personal Characteristics				
1.	Interviewer/facilitator	Which author/s conducted the interview or focus group?	Method / Protocol	
2.	Credentials	What were the researcher's credentials? E.g. PhD, MD	Method / Protocol	
3.	Occupation	What was their occupation at the time of the study?	Method / Protocol	
4.	Gender	Was the researcher male or female?	Method / Protocol	
5.	Experience and training	What experience or training did the researcher have?	Method / Protocol	
Relationship with participants				
6.	Relationship established	Was a relationship established prior to study commencement?	Method / Population and recruitment	
7.	Participant knowledge of the interviewer	What did the participants know about the researcher? e.g. personal goals, reasons for doing the research	Method / Population and recruitment	
8.	Interviewer characteristics	What characteristics were reported about the interviewer/facilitator? e.g. Bias, assumptions, reasons and interests in the research topic	Method / Population and recruitment	
Domain 2: study design				
Theoretical framework				
9.	Methodological orientation and Theory	What methodological orientation was stated to underpin the study? e.g. grounded theory, discourse analysis, ethnography, phenomenology, content analysis	Method / Analysis	
Participant selection				
10.	Sampling	How were participants selected? e.g. purposive, convenience, consecutive, snowball	Method / Population and recruitment	
11.	Method of approach	How were participants approached? e.g. face-to-face, telephone, mail, email	Method / Population and recruitment	
12.	Sample size	How many participants were in the study?	Results	
13.	Non-participation	How many people refused to participate or dropped out? Reasons?	Discussion / Taboo	
Setting				
14.	Setting of data collection	Where was the data collected? e.g. home, clinic, workplace	Method / Protocol	
15.	Presence of non-participants	Was anyone else present besides the participants and researchers?	Method / Protocol	
16.	Description of sample	What are the important characteristics of the sample? e.g. demographic data, date	Results	
Data collection				
17.	Interview guide	Were questions, prompts, guides provided by the authors? Was it pilot tested?	Method / Protocol + Appendix	
18.	Repeat interviews	Were repeat interviews carried out? If yes, how many?	Method / Protocol	
19.	Audio/visual recording	Did the research use audio or visual recording to collect the data?	Method / Analysis	
20.	Field notes	Were field notes made during and/or after the interview or focus group?	Method / Analysis	
21.	Duration	What was the duration of the interviews or focus group?	Results	
22.	Data saturation	Was data saturation discussed?	Method / Analysis	
23.	Transcripts returned	Were transcripts returned to participants for comment and/or correction?	Method / Analysis	
Domain 3: analysis and findings				
Data analysis				
24.	Number of data coders	How many data coders coded the data?	Method / Analysis	
25.	Description of the coding tree	Did authors provide a description of the coding tree?	Results	
26.	Derivation of themes	Were themes identified in advance or derived from the data?	Method / Analysis	
27.	Software	What software, if applicable, was used to manage the data?		No specific software used
28.	Participant checking	Did participants provide feedback on the findings?	Method / Analysis	
Reporting				
29.	Quotations presented	Were participant quotations presented to illustrate the themes / findings? Was each quotation identified? e.g. participant number	Method / Result presentation + Results	
30.	Data and findings consistent	Was there consistency between the data presented and the findings?	Results	
31.	Clarity of major themes	Were major themes clearly presented in the findings?	Results	
32.	Clarity of minor themes	Is there a description of diverse cases or discussion of minor themes?	Discussion	
