# Supplementary material for: General practitioners and sales representatives: Why are we so ambivalent?
Source: PLoS One. 2022 Jan 24;17(1):e0261661. doi: 10.1371/journal.pone.0261661 (PMC8786166; doi:10.1371/journal.pone.0261661)
Supplement: S1 Appendix — (DOC) [file pone.0261661.s002.doc]

# Interview guide

**Sequence of the interview:**

**1 Arrive early, collect first impressions and characteristics of the waiting room**

**2 Test the equipment and start recording**

**3 Thank GP for participation in the research**

**4 Give the general objective of the meeting**

“I'm writing a thesis on information sources. The purpose of this interview is to understand your opinion about the different sources of information available to general practitioners.”

**5 Explain the process and clarify the instructions**

This is an interview, not a questionnaire, so if you don't mind, I'll read you one or more clinical scenarios that will serve as the basis for the discussion.

There are no wrong or right answers. The objective is to obtain a panel of experiences from the doctors I meet.

The interview will be recorded.

Everything said in this interview will be used for research only. The confidentiality of the recordings is guaranteed.

Answer as spontaneously as possible.

If necessary, I will ask you to develop your idea.

I will bring you back to our subject, if necessary, to respect the time frame of the interview.

**6 Start and conduct the interview until the end of the time assigned**

When possible, all questions of the guide should be asked.

**7 Complete the socio-demographic questionnaire and have the consent form signed.**

**8 Immediately after the interview, note:** the interview atmosphere, non-verbal cues, the researcher's impressions and finally the synthesis and key words of the interview.

These scenarios provide a basis to gradually bring up the issue of sales rep visits without causing reluctance and social desirability. Questions from scenario 1 should be asked again after scenarios 2 and 3 if the interviewer feels that the participant’s answers need to be expanded.

**Scenario 1.** Ask to read or read aloud.

1) When you are confronted with a new drug, how do you proceed?

2) What is your experience with congresses? What other sources of information do you regularly use?

3) Can you tell me about the last time you met a pharma sales rep? In general, how do you deal with their information about new treatments?

4) If I understand correctly, sometimes you feel that the pharmaceutical industry is trying to influence your prescriptions... By what means? In these cases, how do you react?

5) So, if I understand correctly, you meet the sales reps and at the same time you are aware that you have to be careful, how do you manage that?

What do you think about this situation? Have you ever considered no longer meeting the sales reps?

6) Do you talk about this with your relatives?

And the colleagues you work with, how do they manage? (Community of influence)

What do patients think about it?

**Scenario 2:**

How did you find out about the properties of Xarelto?

When you are hesitant to prescribe a new or old drug, how do you proceed?

**Scenario 3:**

1) What stance do you take as a GP when you do not agree with the advice given by the specialist?

2) Have you recently had a consultation where you asked yourself: “There is this new drug that I was told about but is it really going to be in my patient's interest?” Can you tell me about this consultation?

3) In fact, I am particularly interested in the apparent contradiction between what you tell me “...” and, at the same time, “...”. How did you come to this opinion?

4) And when you were in medical school, what did you think about it?

**To keep in mind:**

- Renew: “Such as?” “I'm not sure I understood you very well."

- Reformulate.

- Reflective answer: If I understand correctly, on the one hand you want to change but on the other hand you hesitate to drop a source of information.

- Correct me if I am wrong, but it seems to me that your words express a feeling of [guilt, irritation,].

- “How can we tell the difference in this case?”

Scenario 1:

Dr. Michel, a 60-year-old general practitioner, is seeing an 80-year-old man who is confined to a wheelchair with advanced Parkinson's disease. He has been suffering from type 2 diabetes for 15 years and is on metformin 500x3. The patient’s test results show the following:

Fasting glucose 2.3g/l, HbA1c 8.7%, moderate renal failure, ionogram and lipid check-up normal.

Dr Michel has not yet had the opportunity to prescribe Januvia but has already heard about it at a congress and he believes that Mr. Dupont is at risk of hypoglycemia if he prescribes sulfonamides. He seems to remember that it can be prescribed in case of moderate renal failure. After a minute's thought, he decides to prescribe this drug in low doses and perform an HbA1c in 3 months to monitor its effectiveness.

What do you think of this situation?

Scenario 2 :

Dr. Matthieu is being asked by a 60-year-old patient to take care of her mother, an 85-year-old woman who has just arrived in the region to be closer to her family. She is suffering from diabetes and is on metformin and Gliclazide, COPD treated with inhaled corticosteroids and severe Alzheimer's disease with Memantin. She was discharged 1 month ago from the hospital for a proximal pulmonary embolism treated with Warfarin, with an INR fluctuating between 1.5 and 4 since her discharge.

He is reluctant to continue the Warfarin treatment, which he finds constraining, or to switch to Rivaroxaban because he is afraid of an overdose in this multi-medicated elderly patient. Finally, he chooses to adjust the dosage of Warfarin and to reassess the INR in a week.

What do you think of this situation?

Scenario 3 :

Dr. Nicolas, a young doctor, receives Mr. Dupont, 25 years old, in his office for pain in his left leg, without redness or oedema. He finds a painful indurated cord under the skin and diagnoses superficial phlebitis.

He knows that superficial phlebitis can evolve into deep phlebitis and has heard that recent oral anticoagulants can be used for this indication (prevention and treatment of venous thromboembolic events), but he is not too familiar with them.

He decides to make a phone call to a phlebologist friend. The phlebologist tells him: "Now it's much simpler than it used to be. You put him on Rivaroxaban for 10 days and you prescribe an echography. If there is no deep extension you stop after 10 days of treatment and if there is a deep extension you add another 15 days."

What do you think about this situation?

Quantitative questionnaire (sample characterization)

Gender, age, type of area where practicing, additional fees asked.

Year of installation, number of patients in base, SNIR (number of consultations per year), number of working hours per week, practice alone or in a group.

Frequency of receiving pharma sales reps, attending congresses, reading paid journals, free journals, participating in peer groups, other training methods.

Regarding recent drugs, their indications, contraindications and side effects, do you feel uninformed, informed or well-informed?

Regarding the quality of communication with your fellow specialists, do you consider it to be of poor quality, good quality or very good quality?

What about communication with your GP colleagues?

# Consent

I, Adriaan BARBAROUX, a resident in general practice, commit throughout the drafting of my thesis to adopt an ethical posture towards the doctors who will be interviewed, and to maintain a fraternal and respectful attitude.

My work is part of a scientific process of understanding and not of evaluating practices.

I undertake to guarantee the anonymity of doctors. Their names will not be mentioned anywhere in my thesis and I will not transcribe any citations that allow their identification.

By signing this document, the doctors agree to participate in this project and to be interviewed about their medical practice. The interview will be recorded, transcribed and analyzed for the purpose of my thesis only.

Adriaan BARBAROUX, The doctor interviewed:

Resident in general practice
