# Supplementary material for: General practitioners and sales representatives: Why are we so ambivalent?
Source: PLoS One. 2022 Jan 24;17(1):e0261661. doi: 10.1371/journal.pone.0261661 (PMC8786166; doi:10.1371/journal.pone.0261661)
Supplement: Attachment — Submitted filename: 2021 10 08 GPs and sales reps Response to reviewers final.docx [file pone.0261661.s004.docx]

# Response to reviewers:

Dear Barbara Mintzes, Academic Editor,

Dear Joel Lexchin and Lisa Parker , reviewers,

Thank you for giving us the opportunity to improve our manuscript once again. We have considered all your comments carefully. Please find below our point-by-point response.

## Academic Editor reports:

The manuscript currently reads well but there are still some problems with the English. Joel Lexchin has addressed many of the specific changes needed in his comments. However, the manuscript as a whole would also benefit from being read by a native English-language editor and overall stylistic editing. In some cases, 'false friends' are used - words in English that are similar to French words but have nuanced differences in meaning. An example is the use of 'representations' as a results section header and within the subsequent text. You have explained in the text that this term refers to the physicians' perceptions of the sales representative and of their interactions. However, calling this section 'Physicians' perceptions' would be clearer. When representations is used in the text, this could be better replaced by 'perceptions'.

Thank you for this suggestion. A second native reviewer did a final polish of the English. All the changes suggested by Joel Lexchin have been taken into consideration and each occurrence of “representations” have been replaced with “physicians’ perceptions”.

Secondly, Lisa Parker's recommends a shift to how the content in the results and discussion sections, so that all quotes are included as part of the results, in keeping with standard practices for reporting of qualitative research. She also has some suggestions for organisation of content within sections that would make it easier for the reader to understand the key contrasting themes that surfaced in physicians' interviews.

Done (details below in the answer to the reviewer)

Thirdly, table 1 would be more readable at a glance if these data were summarised. For example at the top you could just state n=10 and include one column for all GPs, rather than having a separate column per GP, urban setting could be 6 (60%); Age: Mean (SD); range 30-59; extra fees charged 9 (90%); years in practice mean (SD); range 1-34; enrolled patients mean (SD); range 425-1500; workload (hours per week); mean (SD) etc.

Done. The table now looks like this :

**Table 1. Characteristics of the ten GPs interviewed.**

|  | N = 10 |
| --- | --- |
|  | **(Mean ± SD [Range])** |
| **Age (years)** | 48,8 **±** 9 [30-59] |
| **Years in practice** | 15 ± 11 [1-34] |
| **Enrolled patients** | 906 ± 439 [400-1500] |
| **Hours p. week workload** | 55 ± 8 [43-65] |
| **Number of sales reps p. week** | 3 ± 3 [0-10] |
|  | **(n - percentage)** |
| **Extra fee charged** | 1 (10%) |
| **Urban setting** | 6 (60%) |
| **Group practice** | 5 (50%) |
| **Subscribes to a paid medical journal** | 1 (10%) |
| **Reads commercially-produced free GP journals** | 4 (40%) |
| **Member of GP peer group** | 3 (30%) |

The relevance of some rows (paid and free journal subscriptions) is unclear and for peer group you need an additional description of what the peer group is in the text. I am not sure if these were meant to be indicators of the type of practice the GP has, for example; "Reads commercially-produced free GP journals", or 'Subscribes to La Revue Prescrire' if this is what you were measuring. In the table the row header could be: 'Member of peer GP discussion group'. [or similar] The table should be understandable as a stand-alone item.

Done.

In the comments below, one of Lisa Parker mentions not understanding what you meant by reps not following continuing medical education. I think 'following' may be the problem in this sentence - do you mean that they are not allowed to attend these classes (because of regulations or due to limits imposed by employers?) or that they cannot provide content within continuing education? If this is a comment on a requirement for independence of continuing medical education (e.g. non-sponsorship by industry) this would be an important point.

Indeed, Pharma sales reps are not allowed to provide content for continuing education courses. This sentence was clarified in the text.

## Reviewers' comments:

**Reviewer #1: Joel Lexchin**

The authors have dealt with my major concerns but there remain a number of minor issues, mostly language related, that need to be corrected.

1. Line 64: Rather than "incite" a better word would be "motivate".

Done

2. The material starting "Cognitive dissonance..." on line 67 to the "hidden curriculum" on line 70 does not belong in the Results. It should be moved to the Discussion.

Done

3. The sentence starting "The strength..." on line 70 should be moved to the end of the Discussion.

Done

4. Line 94: Explain what "direct, indirect, and induced effects" means.

Done : “In the United States of America (USA), the biopharmaceutical sector generates nearly $1.2 trillion in economic output annually when direct (biopharmaceutical industry revenue), indirect (vendor and supplier activity), and induced effects (additional private economic activity) are considered(1)”.

5. Line 97: Delete "prescription" and insert "prescribing".

Done

6. Line 98: Delete "highest" and insert "largest".

Done

7. Line 104; Promotes should start with a lower case p.

Done

8. Line 107: I believe that "follow" should be "fund".

Modified as follow : “Pharma sales reps are not allowed to provide content within continuing education courses”.

9. Line 129: Delete "prescription" and insert "prescribing".

Done

10. Line 135: When the authors say "second source" do they mean second most frequently used source or second most trusted source?

The report concluded that, after the drug database *Vidal*, sales rep visits were the second most used source of information (modified)

11. Line 145: Insert "them with" between "provided" and "information".

Done

12. Line 146: Own knowledge of what?

GPs also described a lack of confidence in their own pharmaceutical knowledge when leaving university (modified)

13. Lines 158-159: The authors should explain what they mean by a "constructivist paradigm" and "phenomenological approach".

Done : “An exploratory qualitative study was conducted based on a constructivist paradigm.[25] In this paradigm, qualitative research methods are considered as a means to allow the investigator to be the primary interpretive instrument and reality is considered as socially constructed and knowledge as a product constructed by people taking active part in a research process.[25] An interpretative phenomenological approach was used, that is, individual professional practices were explored and the phenomenon was modeled through the in-depth analysis of singular experiences, the meaning that participants gave to their experience of meeting sales reps, and the underlying psychological mechanisms.[26] The purpose of a phenomenological approach is mainly to shed light on the essence of a person’s experience in relation to a specific phenomenon, in this instance meeting sales reps despite holding a negative opinion of them.”

14. Line 161: When the authors say "this experience" are they referring to interacting with sales reps?

Yes (modified)

15. Line 190: Use “conference” instead of “congress”.

Done

16. Lines 209-210: What is a "paying publication"?

We meant a journal which charges a fee for subscription, this has been modified.

17. Line 210: Delete "to" and insert "in".

Done

18. Line 213: What do "indemnity" and accreditation mean?

Modified as follows : “can give rise to a government payment to members when the group is accredited (registered and recognized by the government)”

19. Line 220: What is a "thesis survey"?

We modified this sentence as follows : “The GPs were asked to answer a survey as part of an end of course medical thesis. The survey was presented to them as dealing with the information sources used by general practitioners, without mentioning promotional detailing visits.”

20. Line 266: Does "verbatims" mean the verbal answers given during the interviews?

In qualitative research, a transcript or verbatim is defined as a textual unit extracted from the transcription of the verbal answers given during the interviews, that can be smaller than a sentence. This has been clarified in the manuscript : “Transcripts, or excerpts of responses, can be grouped into the following categories: motivations, physicians’ perceptions, and values”.

21. Line 299: What sales rep budget restrictions are the authors referring to?

The sentence was unclear: participants referred to budget restrictions that have reduced the influence of the pharmaceutical industry. The sentence was reworded as follows: “The pharmaceutical industry’s persuasive force was described as belonging to a past era because current sales rep budget restrictions mean fewer reps and gifts: “*There’s no more! There’s nothing left!*””

22. Line 364: What do the authors mean by "reassessment"?

We meant self-questioning. Modified.

23. Line 371: Substitute "beliefs" for "cognition".

Done

24. Line 407: Use "companies" instead of "industries".

Done

25. Line 422: Substitute "observations" for "transcriptions".

Done

26. Line 438: Sales visit charter has previously been capitalized.

Thank you for noting this typo.

27. Line 487: It should be “et al”.

Thank you for noting this typo.

28. Line 499: Use "conferences" instead of "congresses".

Done.

## Reviewer #4: Lisa Parker

Major comments

I would have liked to see clearer presentation of the analytic work. At present it seems that the Results section is very descriptive, and the stronger analysis is buried in the Discussion section. I suggest re-ordering some of the text to incorporate the analytic work into Results. This would also adhere more closely to the standard results/discussion format of academic papers in the biomedical sector, where quotes etc are generally not placed in Discussion.

Thank you for this comment, a former reviewer commented that “At the moment, the analysis remains description, but could be greatly strengthened by incorporating the material in the discussion.”, which is the reason we added quotes in the discussion. Following your advice, quotes have been removed from the discussion, as in our former submission.

For example, the authors could consider sectioning the Results into [a] negative views about sales reps [b] reasons for meeting with sales reps (need for quick information; valuing experience-based knowledge; habits acquired from medical school days via the hidden curriculum) [c] how GPs manage the cognitive dissonance (e.g. feeling that self is invulnerable to sales rep tactics etc.) Of course the authors do not have to follow this particular analytic structure, this is just one possible way that the analysis could be taken further to address the stated aim of trying to understand why this phenomenon is happening and what it’s like for the GPs on the ground. I see that the authors have already done a substantial revision in response to previous reviewer comments. Nevertheless, I think a further revision would greatly strengthen the paper.

Thank you for this comment. We modified the structure of the paper to emphasize the concepts and psychosocial mechanisms involved in dealing with ambivalence: they were integrated in the results section, with dedicated subsections for clarity, and we reduced the repetition concerning pragmatism and evidence-based rationale. The hidden curriculum is still in the discussion section, as it relied less on data and can be considered more as explicative of the experience and perspective of participants. There are no longer quotes in the discussion section.

Minor comments

Intro

p 5 line 103 – ‘the information provided by sales reps remains poor’ – what information and who is it provided to ?

Thank you for this comment, we modified this sentence as follows: “the information concerning drugs provided by sales reps to GPs remains poor”

P 5 line 107 ‘pharma sales reps are not allowed to follow continuing medical education courses.’ This comment is not clear to me, what do you mean?

Pharma sales reps are not allowed to provide content within continuing education courses. (modified)

P6 line 137 – ‘this situation is problematic on a financial level … and on a social, ethical and public health level.’ I probably agree with you, but I’d like some more detail here, can you spell out why it is problematic in each of these contexts?

We clarified as follows: “as much as on a social and ethical level (citizens’ demand for more transparency and more independent training for prescribers) and on a public health level because of potentially inadequate prescribing practices.(ref)”

Methods

P 8 line 181 was the preliminary interview data included in this study?

Yes. The data from the preliminary interviews was retained in this study, in accordance with the standards in qualitative research (27,29). (added)

P 10 line 222 you talk about saturation here, but earlier you said that you were not aiming for saturation and it was not needed in a phenomenological study

Indeed, the wording of the sentence could be considered confusing and has been removed. We left the following sentence in the text as a strength concerning the analysis process : “The research team discussed the data sample and considered that it gathered a wide range of experiences about relationships with sales reps and no new theme emerged from the material analyzed”.

P 10 line 230 I would like more information about why ethics approval was not required. Certainly in my country any studies involving human participants requires ethics approval, even when it is a low-risk study such as this one.

Data was collected before January 2016 in France. At this time, ethics approval was needed only for pharmaceutical research. In January 2016, the French “loi jardé” made ethics approval mandatory for some other kinds of research, such as non-pharmaceutical research involving patients. Currently, there is still no legal obligation to ask for ethics approval for this kind of research since the law does not consider qualitative research as research involving human participants. You can find out more at :

<https://www.legifrance.gouv.fr/jorf/id/JORFTEXT000034634217>

« Ne sont pas des recherches impliquant la personne humaine au sens du présent titre les recherches et qui visent à évaluer des modalités d'exercice des professionnels de santé ou des pratiques d'enseignement dans le domaine de la santé. » that we can translate as « Research involving the human person within the meaning of this title does not include research aimed at evaluating the methods of practice of health professionals or teaching practices in the health field. »

Results

P 11 line 253 it’s not useful to give 118 pages of transcriptions unless you provide the font, margins, line spacing etc; you could provide word count? Probably unnecessary; the number and average / median duration of interviews is sufficient I think

We agree with you, this information has been deleted.

Strengths and limitations

P 19 line 446 small number of interviews is not always a limitation of a qualitative study, typically this is how qualitative research works. It would only be a limitation if you were unable to complete as many studies as you wanted to, e.g. if funding ran out or other reasons. In addition it is not always expected to get participants to review their transcripts, and I wouldn’t think this necessarily constituted a limitation unless there were particular reasons e.g. if the participants had strong accents and you were worried about transcribing their words correctly.

Thank you for this comment, we do agree with you. This paragraph was reworded in order not to present it as a limitation.

The research team would like to thank the editor and the reviewers again for the quality of their reviews.

We look forward to hearing from you regarding our revised submission.

Sincerely,

Dr Adriaan BARBAROUX

On behalf of the research team.
